# On Hooping-Cough

**Published:** 1822-12

**Authors:** John Webster

**Affiliations:** Licentiate of the Royal College of Physicians of London, Physician to the Royal Infirmary for Sick Children, &c. &c. &c.


### Art. IV.—*On Hooping-Cough.* By John Webster, m.d. Licentiate of the Royal College of Physicians of London, Physician to the Royal Infirmary for Sick Children, &c. &c. &c.

Of the diseases common to children, none are so frequent, and few so troublesome, as hooping-cough: indeed, such is the nature of this complaint, that parents look forward to its affecting their offspring with much anxiety, and they are not easy on account of their children till it is over; so much so, that it may be said to form an era in the life of the individual, from which many other diseases and affections, that afterwards occur, are dated,—nay, are sometimes even said to arise.

On a complaint so common, and which every mother almost thinks herself fully qualified to treat, it may perhaps be asked if I have any thing new to communicate, to make it the subject of a paper? my only object, then, in this communication is to recommend the trial of a method of cure, which has been lately very extensively employed, under my direction, in a great number of cases of pertussis I have had an opportunity of treating at the institution to which I have the honour to belong; and, as the marked success which has attended the practice usually pursued induces me to think it really beneficial, I am the more anxious to lay it before my professional brethren, through the medium of this Journal. It will be very gratifying to learn the result of the experience and observations of those who may be led to give it a trial; and, should their experience coincide with my own, one step will be made towards the alleviation of a very frequent source of anxiety to parents, and of affliction to the most interesting part of the community. Having this in view will, I am sure, be deemed a sufficient reason for submitting to the public the few imperfect hints contained in this paper; for I am more anxious to communicate what appears to me one useful practical fact, than to compose a regular treatise on this so well known, and already over-written, subject.

Like the history of many other diseases, that of hooping-cough is enveloped in great obscurity, and it is not yet clearly ascertained whether the ancients were acquainted with it or not. The researches which Sprengel has made on this subject tend to show that the disease was first observed by medical writers in France, in the year 1414; since then it has, at various periods, been occasionally epidemic in every part of Europe. In this country it now almost constantly prevails, but is most common in spring and autumn.

When we meet with a child in a violent fit of hooping-cough, the first thing which attracts our attention is the peculiar sound produced during inspiration, the violence of the cough, and the difficulty of breathing: so peculiar, indeed, is this sound, that, once heard, it can never be mistaken; and no other symptom is necessary to be present to constitute the disease. Although this peculiar sound forms the principal (in the vulgar eye the only,) symptom of the disease, pertussis does sometimes occur without the hoop being at all observed. In these cases, the cough is quite different from any other; and, if I may so express it, the sound appears as if it came from some wooden instrument, having a kind of shrillness only to be known by actual observation. Looking at the symptoms now mentioned, a person will naturally conclude that the disease exists principally, if not wholly, in the organs of respiration; and, of course, that all our efforts are to be directed towards the removal of the affection which is thought to exist in these parts. On a nearer examination of the infant labouring under this complaint, we, however, find that the pectoral symptoms are not the only ones that are present, but are preceded or accompanied by pain of the head, and fulness and redness about the temples and eyes; and that these symptoms occasionally give more inconvenience to the child than the cough and difficulty of breathing, which, to a by-stander, appear to constitute the chief part of the disease. To be convinced that this affection of the head is often very severe, we need only observe the blood-shot and inflamed eyes after a fit, and the severe pain which is almost constantly felt in that organ during and after it; the great and decided relief which epistaxis always produces in this disease; and, lastly, the fact that hydrocephalus does sometimes occur as a sequela to this complaint.

That this affection of the head does more frequently occur than is generally imagined, a number of cases I have lately met with fully convince me to be the fact; and I have no doubt, if children could express their previous feelings and symptoms more accurately than they generally do, we should find this to be so. A case which lately came under the observation of my very ingenious and diligent friend, Mr. Webster, of Dulwich, and which he communicated to me, throws much light on the point now in question: it was that of a young woman, a servant in a family where hooping-cough was then prevalent, who also became affected with the disease, not having had it before; but, previous to the cough and hooping manifesting themselves, and before the disease was suspected to be coming on, she complained, for a few days, of great pain and fulness of the head, accompanied with stupor and giddiness. So troublesome and severe were these symptoms, that she was obliged to remain in bed for two days, being unable to move about; and, to use her own expression, "she staggered about the house like one drunk." Many corroborating cases of my own might be adduced, but it appears to me to be superfluous to do so.

As hooping-cough is not now so fatal a disease as formerly, not many opportunities offer themselves to enable medical men to examine into and find out the nature or actual seat of the complaint; consequently, on this point, much obscurity prevails, and many opposite and conflicting opinions are entertained. I believe, however, that, although most men think the primary seat of the disease to be in the organs of respiration, scarcely any morbid change has been found in them; at least, sufficient to prove that the complaint actually began in these organs: and, where disease has been found, might it not have been secondary, and not primary? A great deal has been said about spasm of the air-cells, &c.; but, as I do not exactly comprehend these ideas, and as it serves little purpose to enter further into these and the like speculations, I shall leave them to those who may think they merit attention, or who may better understand what this spasm of the air-cells means.

Considerable difference of opinion exists as to the contagious or infectious nature of hooping-cough: some physicians think that it is so; others, that it is only an epidemic disease. On this point, much discussion has taken place, particularly in Germany, and many names of high reputation are ranked on each side of the question; but, it not being my intention now to give a complete treatise on pertussis, I do not think it necessary further to investigate this fact. At present, I will only remark, that, as far as my own experience goes, I am very much disposed to view this disease in the same light as that of common fever,—namely, that its propagation depends on a particular state of the atmosphere, and of the constitution of the individual affected; and not upon contagion, according to the vulgar meaning of the expression. The circumstance of hooping-cough being very much benefited by a change of air, which, with some, particularly in Scotland, is thought to be the best and only cure, tends much to strengthen this opinion; as also the well-known fact that many children become affected, without ever having had any communication whatever with any children under the disease. At some future period I may, perhaps, attempt to elucidate this point.

Having lately paid a good deal of attention to hooping-cough, and having observed the great influence this affection of the head has upon the disease, I have been thereby led to entertain the opinion that the actual seat of the complaint may be in the head, and that the affection of the respiratory organs is only to be considered as a secondary effect, or as an effort of nature to relieve herself by expanding the lungs to an unusual degree, thereby allowing a greater quantity of blood to flow into them, which may in some degree diminish the fulness and congestion now spoken of. This is a very mechanical theory, but it appears to me not altogether inconsistent, and is somewhat plausible. To the opinion that the seat of hooping-cough is in the head, I have been led from being informed, by my very intelligent and experienced friend, Mr. Wardrop, that he had found, on examining the head of an infant who died of pertussis, very evident marks of congestion and inflammation of the brain and its membranes; and that he thought blood-letting and leeches would have been very useful in this case. Some fatal examples of the same kind, which I have had an opportunity myself of examining, in which there were both signs of previous inflammation and congestion, and one with more water than usual in the ventricles,—also the circumstance that, generally, no marks of disease are found in the lungs,—strengthen this opinion. Besides, every one must often have observed the great relief epistaxis gives in this complaint; and every body also knows, where the child bleeds profusely at the nose, the disease is generally more mild than otherwise; for this is nothing more than, as diarrhœa is in the beginning of fever, an effort of nature to cure the disease. Further, if any reasoning can be allowed from the success of particular treatment, certainly the result of the plan I have lately pursued, and which is nothing more than simply doing by art that which nature occasionally does of herself, confirms me in the opinion I have lately taken up, and have endeavoured to point out and to elucidate. However, as I may be mistaken, and as I should wish to have more experience and morbid examinations, either of my own or of others, before boldly asserting as a fact what I now suspect to be the case, I shall pass on to detail the treatment I employ, which, whatever may be the pathology of hooping-cough, certainly has, in my hands, been most successful: it is simply to follow the dictates of nature, *by relieving this congestion in the head* that has been already shown to exist; whether primary or secondary, it remains for future experience and investigation fully to establish.

The best way to carry this object into effect, is to apply, as will be naturally concluded, leeches to the forehead and behind the ears; for there the blood being drawn from parts supplied by the ramifications of the supraorbital arteries, and of those of the internal ear, the blood is drawn more directly from the head than it would be were the leeches applied to the temples. This observation will be found fully exemplified in cases of common fever with a determination of blood to the head, where leeches applied to the forehead do often a great deal more good than when applied to any other part.

Upon the same principle, the jugular vein may be opened with much advantage. I have not myself had recourse to it in any case, because I have always got enough blood in the way already mentioned: besides, as the children of the poor are very likely to be neglected, I would not wish to trust to the parents, least they should, from carelessness, allow the bandages to slip off, and much blood might thereby be unnecessarily lost. However, I have no doubt but that it would be very useful; and, in private or hospital practice, I would have recourse to it in obstinate cases.

General blood-letting is also very beneficial; and, if we look into some of the older, as also the modern, writers, we shall find it very much recommended, particularly if the patient be of a full inflammatory habit of body, although the theory which led to its employment be different from the one here advanced. It is a remedy which may be had recourse to with advantage, especially where the patient is plethoric or affected with any general febrile action; the leeches being also employed for the removal of the head-ach and other symptoms.

In addition to the evacuation of blood by applying leeches to the forehead, I keep the bowels open, by moderate and frequent doses of calomel and rhubarb, or calomel and ipecacuanha, and give squill and antimonial mixtures for the cough; but these remedies are of secondary importance, and only used as occasion requires: the leeches are what I principally, and often entirely, depend on.

As cases sometimes occur where, from the long continuance of the disease, and the consequent exhausted state of the little patient, any loss of blood would not be advisable or prudent, I then substitute blisters behind the ears and to the nape of the neck, and give digitalis: this medicine, besides its peculiar action on the heart and arteries, has also a *local effect* (if I may so express it,) in diminishing the determination of blood to the head; and I am inclined to think so, as well from the opinion of the celebrated Professor Borda, of Pavia, as from having observed its great efficacy in the cure of diseases in adults accompanied with a disposition to fulness of blood in the head; it may, therefore, be used in those cases of hooping-cough where any loss of blood is not thought prudent.

The success I have met with in the treatment of pertussis may, perhaps, not be altogether credited; but I would only request practitioners to make trial of the method of cure now recommended, to see its efficacy. I have been often very much gratified, nay, even surprised, at the great, and often immediate, relief which the application alone of a few leeches to the forehead has produced. I have frequently met with children affected with this malady for four or five weeks, come back a few days after the leeches were employed, and their parents tell me that the hooping ceased soon after the blood had been evacuated, and that they now only had a little cough; which also generally yielded, in a few days more, to the use of purgatives and saline and antimonial medicines, or to a second application of the leeches.

Here it may not be uninteresting to observe, that many medical men are of opinion that pertussis, like many other contagious diseases, must run through a certain course; and, consequently, that little can be done either to check its progress or to cut it short altogether; and all that we can hope to accomplish is to be able to moderate its violence. The opinion that little can be done in this disease, I am very much disposed to hold not only as highly detrimental to the patient, and a complete bar to farther improvement in medical science, but as erroneous, although supported by the great authority of Sydenham, Werlhof, Hufeland, &c. My own experience has fully convinced me that this disease may be cured at any period of its course; and that it is only our ineffectual practice, not the disposition of the malady to go through certain stages, which prevents its being more frequently cured than it generally is. In this, as in every other complaint, no pre-conceived opinions as to its incurable nature, or its disposition to run a certain course, ought to influence or to paralyze our exertions: it is quite unscientific for a medical man to be thus influenced, and is besides unfeeling, and throws discredit on medicine to allow that nothing can be done.

At the Infirmary for Sick Children, I have employed the treatment now recommended, during the last eight months, in one hundred and eleven cases, independent of many others which I have otherwise had an opportunity of seeing; and I can, without any exaggeration, assert that the application of leeches to the forehead has been followed by almost immediate and very marked success; and I would advise their employment to others, trusting that the practice will be as useful in their hands as it has been in mine. So common is it with me to have recourse to this remedy, that the mothers, when they bring their children to the institution, have more than once asked me if they should apply leeches or not? and I have met with several instances where the parent told me that she would keep the leeches by her, ready to use, should the little patient again have a relapse, so convinced was she of their utility.

To enter into a detail of cases successfully treated in the manner now mentioned, I conceive to be totally unnecessary; it would only swell this paper to a tiresome length. The particular effect of treatment, or the general observations they suggest, are all that is wanted: the more so, as every man can easily, by his own experience, investigate the truth of what I have advanced, by a reference to the cases which may come under his own observation.

To conclude:—it affords me much satisfaction to be able to add, that my friend, Mr. Webster, of Dulwich, whose assiduity in the pursuit of knowledge and attention to the study of his profession is worthy of praise, has been induced, from observing the success of this treatment at the Infirmary for Sick Children, to give it a trial in his practice; and he informs me that he has done so, in eleven cases, with very decided benefit. In some, the disease was cut short in a week or ten days, which otherwise, in every appearance, would have continued for a month or two. One family, in particular, where three children, of full plethoric habits of body, were severely affected with hooping-cough, showed clearly the utility of this treatment.

One of the children, from twelve violent fits of a night, was, two days after the application of these leeches to the forehead, (taking at the same time a purgative mixture,) only five times affected; the leeches were applied a second and a third time, when the boy had only one fit during the night: in a few days afterwards he was free from all complaint. The other two cases did equally well.

*October 10th, 1822.*

